# Outstanding Reviewers for *RSC Chemical Biology* in 2020

**DOI:** 10.1039/d1cb90016a

**Published:** 2021-05-25

## Abstract

We would like to take this opportunity to highlight the Outstanding Reviewers for *RSC Chemical Biology* in 2020, as selected by the editorial team for their significant contribution to the journal.
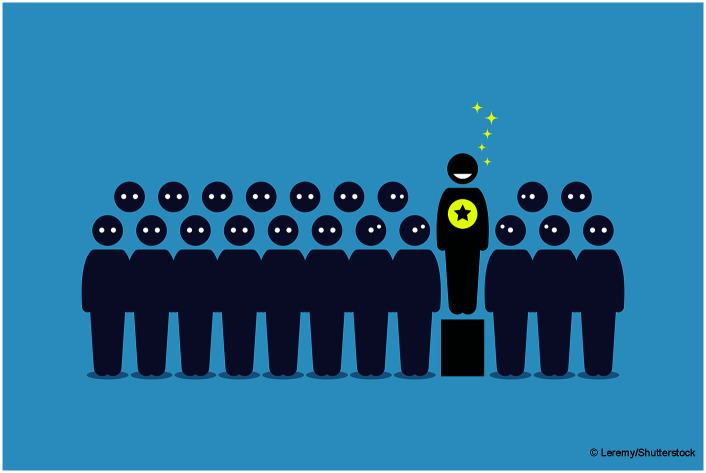

We would like to take this opportunity to thank all of *RSC Chemical Biology*’s reviewers, and in particular highlight the Outstanding Reviewers for the journal in 2020, as selected by the editorial team for their significant contribution to *RSC Chemical Biology*. We announce our Outstanding Reviewers annually and each receives a certificate to give recognition for their contribution. The reviewers have been chosen based on the number, timeliness and quality of the reports completed over the last 12 months.

 

Dr Marco Di Antonio

Imperial College

ORCID: 0000-0002-7321-1867

 

Dr Herman Overkleeft

Leiden University

ORCID: 0000-0001-6976-7005

 

Dr Chu Wang

Peking University

ORCID: 0000-0002-6925-1268

 

We would also like to thank the *RSC Chemical Biology* Editorial Board and Advisory Board and the chemical biology community for their continued support of the journal, as authors, reviewers and readers.

 

Dr Anna Rulka, Executive Editor

## Supplementary Material

